# A Modeled Analysis of Telehealth Methods for Treating Pressure Ulcers after Spinal Cord Injury

**DOI:** 10.1155/2012/729492

**Published:** 2012-08-28

**Authors:** Mark W. Smith, Michelle L. Hill, Karen L. Hopkins, B. Jenny Kiratli, Ruth C. Cronkite

**Affiliations:** ^1^Truven Health Analytics, Washington, DC 20008, USA; ^2^Center for Primary Care and Outcomes Research, Stanford University, Stanford, CA 94305-6019, USA; ^3^Spinal Cord Injury Center, Veterans Affairs Palo Alto Health Care System, Palo Alto, CA 94304, USA; ^4^Center for Health Care Evaluation, Veterans Affairs Palo Alto Health Care System, 795 Willow Road (152 MPD), Menlo Park, CA 94025, USA; ^5^Department of Sociology, Stanford University, Stanford, CA 94305-2047, USA

## Abstract

Home telehealth can improve clinical outcomes for conditions that are common among patients with spinal cord injury (SCI). However, little is known about the costs and potential savings associated with its use. We developed clinical scenarios that describe common situations in treatment or prevention of pressure ulcers. We calculated the cost implications of using telehealth for each scenario and under a range of reasonable assumptions. Data were gathered primarily from US Department of Veterans Affairs (VA) administrative records. For each scenario and treatment method, we multiplied probabilities, frequencies, and costs to determine the expected cost over the entire treatment period. We generated low-, medium-, and high-cost estimates based on reasonable ranges of costs and probabilities. Telehealth care was less expensive than standard care when low-cost technology was used but often more expensive when high-cost, interactive devices were installed in the patient's home. Increased utilization of telehealth technology (particularly among rural veterans with SCI) could reduce the incidence of stage III and stage IV ulcers, thereby improving veterans' health and quality of care without increasing costs. Future prospective studies of our present scenarios using patients with various healthcare challenges are recommended.

## 1. Introduction

Telehealth, or telemedicine, is the use of telecommunications and information technology to provide health care when distance separates the participants. A number of studies have shown that home telehealth interventions can improve clinical outcomes for a variety of conditions such as dermatology [1] and diabetes [2–4]. Several studies have investigated its use in persons with spinal cord injury (SCI), in particular for the ability to detect and stage pressure ulcers (PUs) [5, 6]. Multiple intrinsic and extrinsic factors make these ulcers common in patients with SCI. For example, transfer-mobility problems, home-environment adaptive equipment issues, recreational activities, spasticity, and bowel and bladder incontinence may all contribute to the occurrence of skin compromise and PU development. Telehealth appears to be a logical option for PU diagnosis and followup, particularly when the patient is not close to a local facility or transportation is difficult. However, there has been no investigation into the costs and potential savings associated with its use for persons with SCI in the US Department of Veterans Affairs (VA) health care system. This is unfortunate because of the relatively large number of patients with SCI in VA facilities.

The VA has developed a network of major SCI rehabilitation sites, which are often called *hubs*, and has identified smaller VA facilities in the same Veterans Integrated Service Network (VISN), which are often called *spokes*. The *spokes* can consult with the major facilities on SCI care [7]. To support this effort, the VA's headquarters has provided a significant amount of telehealth equipment to *hub* and *spoke* sites. What has been lacking is explicit guidance on when to use it. Certain VA medical centers have instituted their own telehealth programs [8, 9], but in general the efforts have not been coordinated. From a management perspective, there has been little guidance about its clinical impact and none with respect to cost. 

Telehealth consultation between hospitals could improve patients well-being while saving money and increasing access to expert care. In one common situation, a veteran with SCI and an apparent PU presents at a hospital that lacks an SCI Center. The clinicians see few patients with SCI and feel that they need consultation with a specialist. The patient is then transported in a specialized ambulance to the nearest *hub* site. This trip often takes several hours and may require an overnight inpatient stay if the return cannot be made during normal hours. If a telehealth connection was available between facilities, the expert consultation could occur without the inconvenience and potential discomfort of transporting the patient. Transportation can actually contribute to the PU through an extended period sitting or lying down. The VA could reduce costs and, depending on his/her copayment status, the patient may also save money. 

A similar situation arises when patients are treated at home. Standard or usual care often includes home visits in order to check for PUs and/or the status of any ulcers already under treatment. Telehealth technology installed in the home could reduce the need for such visits. The options range from simple hand-held cameras to large machines that enable live interaction with clinicians. The existence of both high-cost and low-cost telehealth technologies suggests the need for an analysis of costs and outcomes. 

### 1.1. Objectives

The present study has two major objectives: (1) to develop clinical scenarios that describe common situations in the treatment or prevention of PUs, and (2) to estimate the cost implications of using telehealth under each scenario by using a range of reasonable assumptions. We allow clinical outcomes to vary between scenarios with and without telehealth by varying the likelihood that patients will need inpatient and outpatient care of various kinds. The results allow for a systematic assessment of the relative costs of telehealth compared to usual care for PUs among veterans with SCI. 

## 2. Methods

### 2.1. Phase 1: Development of Clinical Scenarios

 In Phase 1, clinical experts in SCI care developed clinical scenarios that represent common patient situations. Each scenario depends on whether the patient resides within (100 miles or a two-hour drive) or outside the SCI homecare catchment area. We describe typical care without telehealth and different alternative care options with telehealth. The scenarios assume that the patient is in a private home residence that includes a caregiver, or in a skilled nursing facility. *SCI Center *refers to a specialized SCI treatment center at a VA *hub* facility. 

For reference, the four stages of pressure ulcers are explained in Table 1. Stages I and II represent closed wounds and are treated on an outpatient basis. Stages III and IV represent open wounds. Stage III pressure ulcers may be treated in an inpatient setting depending on the precise clinical situation of the patient, while stage IV ulcers always require inpatient care.

The characteristics of each scenario and option are summarized in Table 2 and briefly described below. The specified frequency and length of the healthcare visits are estimated based on customary recommendations; the times may be adjusted according to patient needs.

#### 2.1.1. Patient Scenario 1: Primary Prevention for a Newly Injured Patient Discharged to Home from an SCI Center after Rehabilitation and without a PU

**Within SCI Homecare Catchment**

*Usual care* for patients within the homecare catchment is for a registered nurse (RN) from the SCI homecare staff to visit the patient in his/her residence for preventative, educational, and monitoring purposes. Patients typically return to the SCI Center for an outpatient visit and/or an inpatient stay for a 1-year annual follow-up examination.

*Home telehealth care option 1* involves the use of a videoconferencing unit that is attached to a land-line telephone as a complement to standard homecare visits. The telehealth unit is placed in the patient's home or in a nursing care residential home (which could serve more than one patient). The videoconferencing unit allows the SCI homecare practitioners to substitute 50% of the usual care visits with home telehealth visits.

**Outside SCI Homecare Catchment**

*Usual care* for a patient who resides outside of the SCI homecare catchment boundary is follow-up telephone contact at 3 and 6 months after discharge. An in-person follow-up examination at the SCI Center takes place at 12 months. The patient is also advised to visit a local outpatient clinic or VA medical facility near his/her residence for any care, as needed. This scenario assumes that the patient does not have any active diagnoses upon discharge. The PU itself may be treated if the patient seeks care for skin or PUs at a non-SCI specialty clinic or facility, but the contributing factors and intricacies of SCI specialty care may not be adequately addressed. This lack of SCI expert care may impact the successful treatment of existing wounds and may not be optimal for preventing additional skin compromise.

*Home telehealth care option 1* uses the same videoconferencing unit and the same schedule as option 1 for patients within the homecare catchment. The unit allows the practitioners to interact and assess the patient on a regularly scheduled basis and via home telehealth visits as needed. A patient telehealth unit is placed in the patient's home or in a nursing home setting. 

*Home telehealth option 2* is designed to leverage the SCI Center expertise through the *hub and spoke* model of care. The patient is sent home with the same videoconferencing unit as per telehealth scenario 1 and follows the same schedule. In this scenario, the patient connects with the SCI center; this provides oversight by the SCI Center with appropriate clinical attention and intervention as identified via patient response. In addition, the patient has a planned visit with a local SCI consultation clinic (a local *spoke* VA hospital or outpatient clinic) at 1, 3, 6, and 9 months after discharge. The local clinic *spoke* would connect via videoconferencing with the SCI Center clinician. The patient and clinician are present at the *spoke* clinic. 

#### 2.1.2. Patient Scenario 2: An Established SCI Patient Diagnosed with a PU That Requires Dressing Changes Until Healed

**Within the SCI Home Care Catchment**

*Usual care* for a patient with one or more PUs is regular home visits by a homecare RN. The nurse changes dressings and takes a digital photo for the medical record and potential physician review. There is currently *no telehealth option* for this situation.

**Outside the SCI Home Care Catchment**

*Usual care* is for a patient to be managed by a contracted private home care agency or admitted to a skilled nursing facility until the PU is healed. In the model below, we assume that an agency is used.

*Home telehealth option 3* involves *store-and-forward* telemedicine integrated with telehealth consultation. The patient is managed by a homecare agency or nursing facility as in usual care. A digital camera and instruction kit are provided to the caregiver or nurse. If it is the caregiver, we assume that he or she is willing and able to operate the digital camera. This option also assumes that secure internet access is available for transmitting the photos via e-mail. Digital photos of the PU and surrounding skin are taken every week. They are then forwarded to the SCI Center for review by a nurse and for documentation in the medical record. The nurse contacts the managing agency for any treatment recommendations. SCI telehealth consultation with a nurse practitioner or physician at a local VA *spoke* facility is scheduled if the wound is either getting worse or appears healed. In case of poor healing or complications, expeditious intervention and treatment plan alterations would be implemented. 

#### 2.1.3. Patient Scenario 3: Prevention of Recurrence after Surgical Treatment

**Within the SCI Home Care Catchment**

*Usual care* for a patient discharged after a two-month inpatient stay that included plastic surgery to repair a PU involves one return visit to the SCI outpatient clinic after one month to recheck the site and follow-up visits as needed.

*Telehealth model of care option 1* incorporates both home telehealth and store-and-forward telehealth. After discharge to home, the patient videoconferences with a nurse using the same home unit previously described. Digital still photos of the surgical site are taken using the telehealth equipment (rather than a digital camera) during a telehealth visit. These photos are forwarded into the medical record and made available for the plastic surgeons to review, if a consultation is requested by the SCI Center staff. This model works for patients regardless of the distance of their residence from the SCI Center.

*Telehealth model of care option 3* substitutes a digital camera for the home video telehealth unit. Digital cameras provide a greater resolution (3.3 megapixels minimally required) than a video still-shot and are the standard for documenting skin and plastic surgery repairs. The photo is taken and forwarded to the SCI Center for review and incorporation into the electronic medical record.

**Outside the SCI Home Care Catchment**

* Usual care* is the same except for followup. If the patient lives over 100 miles or two hours from the nearest SCI Clinic, care is provided by a VA medical center or other facility near the patient's home.

*Telehealth model of care option 1* incorporates both home telehealth and store-and-forward telehealth. After discharge to home residence, the patient videoconferences with a nurse. In addition, digital still photos of the surgical site are taken using the telehealth equipment. These photos are forwarded for incorporation into the medical record and made available for the plastic surgeons to review, if a consultation is requested by the SCI Center staff. If the patient and surgical site require further assessment, a telehealth consultation can be scheduled for those patients living over 100 miles or two hours from the SCI Center. The telehealth consultation would take place between a patient at an SCI *spoke* site and clinicians (including plastic surgery specialists) at the SCI Center *hub* site. 

*Telehealth model of care option 3* incorporates utilizing digital cameras in place of the home video telehealth unit and is the same as option 3 within the SCI home catchment. If the patient and surgical site require further assessment, a telehealth consultation can be scheduled with the SCI center. The telehealth consultation would take place between a patient at an SCI *spoke* site and clinicians at a *hub* site.

### 2.2. Phase 2. Estimation of Costs

In Phase 2, we determined the cost of each scenario under usual care (without telehealth) and under alternative scenarios that included telehealth technology options. Probabilities of developing ulcers came from the expertise of the clinicians on the project. Most figures were drawn from VA administrative data. About 20,000 individuals are treated for SCI in the VA system each year. Of these, roughly half will live in the catchment area of a VA medical center. About one-third (32%) will be eligible for telehealth; the remainder either lack sufficient functional independence (20%) or lack a standard land-line telephone (48%).

**Costs Associated with VA Care**

Costs were extracted from the VA Decision Support System (DSS) National Data Extracts (NDEs). The DSS allows for the estimation of costs for every inpatient and outpatient VA encounter. Information on costs of VA-funded care for home-based health care and for care at certain non-VA facilities, such as rehabilitation hospitals and community nursing homes, was extracted from the VA Fee Basis program files.

**Costs Associated with Telehealth**

A second set of VA costs pertained to the telehealth system. Its elements included equipment, training, and telecommunication line costs. Equipment costs were found in the Federal Supply Schedule and from VA staff in the Acquisition and Material Management Service. Training costs were based on national-average VA staff costs in a technical report from the Health Services Research and Development (HSR&D) Health Economics Resource Center [11]. Other supply costs, such as for telecommunication lines, were drawn from published studies [12, 13].

**Miscellaneous Costs**

There were also several non-VA costs to estimate. These included travel costs under various modes of transportation and the cost of any paid home caregiver. We used the IRS standard mileage reimbursement rate for car travel. We estimated the costs of other modes of transportation through internet research of private firms providing transportation. Home caregiver costs were estimated using average national wage rates for such care, as determined by the US Department of Labor, Bureau of Labor Statistics. A second source of salaries was the web site http://Salary.com/.

**Cost Analysis**

For each scenario and treatment option, we multiplied probabilities, frequencies, and costs to estimate the cost over the entire expected treatment period. We generated low-, medium-, and high-cost estimates based on reasonable ranges of costs and probabilities.

### 2.3. Model Inputs

Improving medical care can have secondary impacts on the VA health care system. The prevalence of SCI is relatively low, and we assumed that the availability of telehealth would not be a sufficient incentive for patients with SCI to enroll in the VA health care system if they had not done so earlier. Consequently, demand for SCI care only among current SCI patients was explicitly modeled here. We assumed no change in demand for other conditions because there was no guidance for predicting such changes and because the variation in changes could be large. Finally, we assumed no changes in staffing at the VA as a result of telehealth use.

We present costs associated with each scenario, both for standard care without telehealth and for telehealth-enhanced care. We did not conduct any statistical tests comparing the costs across scenarios because these are modeled costs rather than averages from individual observations. Unit costs appear in Table 3.  Table 4 presents the base estimates and sensitivity ranges for prevalence rates in the model.

**Telehealth Costs**

Digital cameras range in cost from $200 to $300 and are assumed to last three years. The home videoconference machine commonly used in the VA in 2007 was the American TeleCare LifeView machine (Eden Prairie, MN). Its cost of $11,325 came from a national contract with the VA and hence has no variation. We assumed that it lasts three years. Telephone calls and the LifeView station both use telephone land lines. Based on actual experience, we expected that 52% of individuals eligible for telehealth would have standard (land-line) telephones. No cost was assigned for them because this program does not purchase or repair telephones, and their use would not noticeably shorten their lifetimes. We assumed that the VA clinicians initiated all calls in order to eliminate any cost to the patient. Based on our assumption that transmission costs were part of the fixed overhead assigned to encounters in the SCI clinic, we did not account for their costs separately.

**Home-Based Care Encounters**

There were three types of home-based care encounters: one with a VA registered nurse (RN), one with a contract RN, and one with a VA nurse or doctor at an SCI clinic via the LifeView machine. Nurse wages in the VA are not unusually high, but the costs for VA nurses were more than five times those for contract nurses (Table 3). We conclude that the difference stems from overhead costs in the SCI service of VA medical centers. Finally, we assumed that using a digital camera would not lengthen the time it takes to examine a patient for PUs, and thus did not account separately for the cost of using a digital camera.

**Facility-Based Encounters**

There were five types of facility-based encounters. The first three reflect encounters at VA hospitals. The fourth (telehealth call) includes the patient and a hospital-based staff member. The fifth refers to office visits by contract physicians. VA payments to office-based physicians are often similar to Medicare payments for the same encounters. The relatively low cost for the contract physician visits reflects the lack of a hospital facility component in the payment.

**Inpatient Care**

There were two types of inpatient care. The first was plastic surgery and all inpatient recovery following detection of a severe PU. The second was the 30-day average cost of VA payments to community skilled nursing facilities (SNFs). In our models, the time spent in SNFs was measured in 30-day increments. Although the VA has its own SNF units, most VA convalescent patients are treated in community facilities. This is particularly true for individuals living far from a VA hospital.

**Transportation**

Transporting persons with SCI requires wheelchair accessibility. The *low* estimate reflects only mileage costs and assumes that the patient uses a private vehicle. The *medium* and *high* estimates reflect a reasonable range of costs for private transportation by a medical transportation firm.

## 3. Results

Cost results for each scenario appear in Table 5. Overall, we found that telehealth care was less expensive than standard care when patients and facilities used low-cost technology, (e.g., digital cameras and e-mail). Telehealth was more expensive when high-cost, interactive devices were employed in the patient's home.

For primary prevention of PUs, telehealth care using the advanced, interactive technology (telehealth option 1) was slightly less expensive than usual care in the first year. It was also less expensive in later years (figures not shown). Telehealth support of a *spoke* site by a *hub* site (telehealth option 2) was more expensive than usual care in the first year but slightly less expensive in later years. 

In the scenario of conservative treatment for an existing ulcer, telehealth care is not available for persons within the VA catchment area. For those outside the area, telehealth care using a digital camera (telehealth option 3) was slightly less expensive than usual care under all cost assumptions. 

For postsurgical care, the telehealth approach with an interactive system in the user's home (telehealth option 1) was substantially more expensive than usual care in every case except one. Conversely, a telehealth method that relied only on digital cameras (telehealth option 3) was notably less expensive than usual care. 

Care within the SCI homecare catchment area was generally more expensive than care outside that area because of the relative cost of the nursing staff. As noted earlier, although labor costs for VA nurses are not high, the overhead assigned to nurses in the SCI service of VA hospitals led to a total estimated cost well above that of contract nurses. 

## 4. Discussion

### 4.1. Clinical Implications

Pressure ulcers are a common and costly problem among persons with SCI. Videoconferencing is a valid method for recognizing and staging PUs [6, 14]. In the present study, we analyzed costs across a range of clinical scenarios and found that low-cost telehealth strategies could reduce the cost of care for persons with SCI. The models imply that patients such as rural veterans with SCI may be able to obtain clinically effective and cost-effective care for PUs through inexpensive telehealth technology. An increase in use of telehealth technology could reduce the incidence of stage III and stage IV ulcers, thereby improving veterans' health without increasing VA costs. 

### 4.2. Cost Implications

Using the model results, we calculated the yearly system-level cost implications of employing the telehealth approaches relative to usual care.  Table 6 presents the range of net savings (positive numbers) or net costs (negative numbers) for each scenario, within and beyond the catchment areas. To summarize, in scenario 1 (preventive care) telehealth approaches were cost-saving within the homecare catchment area but not always outside of it. In scenario 2 (treatment of an existing PU), telehealth was cost-saving outside the homecare catchment area. Note that there is no telehealth within the homecare catchment area for scenario 2. For scenario 3 (postsurgical care), telehealth approach 1 was always more expensive while approach 3 was always cost-saving. 

The last two rows of the table present ranges of the total net savings if the VA were to adopt telehealth care for SCI whenever it was feasible. If the VA relied entirely on telehealth approach 1, then it would face higher expenditures unless the highest-cost assumptions prevailed. However, using alternative, lower-cost telehealth approaches where possible would enable the VA to find net savings in the millions of dollars under low-, medium-, or high-cost assumptions.

### 4.3. Limitations and Future Research

 We analyzed the cost of SCI care through a model that used average costs and estimated probabilities of clinical outcomes and equipment failure. As with any study, actual costs and outcomes will vary by person and by VA station. Our results should be interpreted in light of the uncertainty that comes from using average figures and assumed probabilities.

We reflected differences in clinical outcomes with and without telehealth technology by altering the probability that patients would heal within a specified timeframe, and the related probabilities of needing inpatient and outpatient care. These estimates reflect the experiences of a single VA site and might not generalize to the entire VA system.

The use of low, medium, and high costs constitutes a simple form of multiway sensitivity analysis that indicates the direction of difference (positive or negative) between standard and telehealth approaches. A more complete analysis would also vary the underlying probabilities. The relative cost of SCI care with and without telehealth will naturally depend, at least in part, on differences in clinical outcomes and the VA's savings or costs.

Considerable research remains to be done in the area of telehealth care for SCI. Topics could include tracking actual costs for use of specific telehealth technologies by a well-described population, such as persons enrolled in a clinical trial. It would also be beneficial to track the clinical and cost outcomes of particular telehealth technologies in different settings and with different types of patients. In other words, it would be useful to replicate our present scenarios with prospective studies.

## Figures and Tables

**Table 1:** Pressure ulcer stages and attendant dangers.

Stage	Description/dangers
Stage I	Intact skin with nonblanchable redness of a localized area usually over a bony prominence. The area may be painful, firm, soft, warmer, or cooler compared to adjacent tissue. May indicate persons at risk of ulcer progression

Stage II	Partial-thickness loss of dermis presenting as a shallow open ulcer with a red-pink wound bed, without slough (necrotic tissue). May also present as intact or open serum filled blister. May progress to stage III if pressure is not relieved

Stage III	Full-thickness tissue loss. Subcutaneous fat may be visible, but bone, tendon, and muscle are not exposed. Slough may be present but does not obscure the depth of tissue loss. May include undermining or tunneling. May progress to stage IV if pressure to wound area is not relieved

Stage IV	Full-thickness tissue loss with exposed bone, tendon, or muscle. Slough may be present on some parts of the wound bed. Often include undermining and tunneling. Osteomyelitis (infection of the bone) may develop in wounds with exposed bone

Source: National Pressure Ulcer Advisory Panel (NPUAP).

**Table 2:** Characteristics of the usual care and telehealth care programs that were investigated.

	Healthcare program			
	Usual care (without telehealth)	Telehealth option 1	Telehealth option 2	Telehealth option 3
Scenario 1: Preventive care				
Within SCI homecare catchment area				
Method	Home visit	Telephone system video-conference unit connected with clinician		
Frequency of visits	1 x/wk for 3 mo; 2 x/mo for 9 mo; patient visits SCI Center at 1 year	Substitute 50% of usual care visits with telehealth		
Clinician time per visit	120–360 min with travel time	30 min		
Outside SCI homecare catchment area				
Method	Telephone contact with clinician and SCI Center visit; local outpatient facility visits as needed	Same as within homecare catchment	Telephone system video-conference unit connected with patient, local facility, and SCI center	
Frequency of visits	SCI Center at 3 mo and 1 year; call at 6 mo	Same as within homecare catchment, includes SCI Center visit at 1 year	Same as within homecare catchment; *plus* patient visits local clinic, which teleconnects with SCI Center, at 1,3,6,9 mo	
Clinician time per visit	30 min	30 min	45 min	

Scenario 2: Existing pressure ulcer				
Within SCI homecare catchment area				
Method	Home visit: change dressing; take photo	No telehealth options		
Frequency of visits	1 x/wk, as needed			
Clinician time per visit	240 minutes, includes digital photo			
Outside SCI homecare catchment area				
Method	Contract with private nursing agency^a^			Provide digital camera to caregiver or nurse; photos sent to SCI center
Frequency of visits	1 x/wk until healed			1 x/wk until healed
Clinician time per visit	30–60 min			10 min to review photo; 30–45 min if videoconference with local facility is needed

Scenario 3: Postsurgical care				
Within SCI homecare catchment area				
Method	Homecare visits	Video-conference unit connected with clinician plus digital camera for patient or caregiver		No video-conference unit; digital camera to patient or caregiver only
Frequency of visits	By 1 mo, then 1-2 x/mo for 3 mos; additional SCI outpatient care as needed	2 x/mo for 3 mos		2 x/mo for 3 mos
Clinician time per visit	120–360 min	30 min		10 min
Outside SCI homecare catchment area				
Method	Patient visits nearest VA facility	Telephone system video-conference unit connected with patient, local facility, and SCI center		Same as within homecare catchment area
Frequency of visits	At 1 mo; additional SCI outpatient care as needed (not an SCI Center)	1-2 x/mo for 3 mos		1-2 x/mo for 3 mos
Clinician time per visit	45–60 min	30 min		10 min

^a^An alternative to hiring a contract nurse, not modeled here, would be to admit the patient to a skilled nursing facility.

**Table 3:** Inputs to the cost model.

	Unit cost estimates^a^		
	Low	Medium	High
Staff costs (per hour)			
Registered nurse (VA staff)	$33	$37	$40
Registered nurse (contractor)	$50	$55	$60
Physician (VA staff)	$108	$120	$132
Equipment (per year)			
Standard telephone^b^	$0	$0	$0
Digital camera	$67	$83	$100
Home videoconferencing	$3,775	$3,775	$3,775
Transmission cost^c^	$0	$0	$0
Home encounters (each)			
VA RN visit	$549	$610	$671
Contract nurse visit	$109	$118	$127
Interactive telehealth call	$227	$252	$277
Facility encounters: outpatient (each)			
Large SCI clinic (*hub*) visit	$259	$630	$816
Small SCI clinic (*spoke*) visit	$436	$484	$532
*Hub*-*spoke* conference	$668	$742	$816
Telehealth call with patient	$84	$93	$102
Contract physician office visit	$44	$55	$66
Facility encounters: inpatient			
VA surgery and recovery (total)	$38,875	$73,049	$218,067
Community SNF (30 days)	$7,503	$8,337	$9,170
Transportation (round-trip)			
Wheelchair-enabled transport	$6	$138	$274

RN: Registered nurse; SCI: spinal cord injury; SNF: skilled nursing facility; VA: U.S.  Department of Veterans Affairs.

^a^Figures are in 2007 dollars.

^b^Standard telephones are already available and no meaningful cost from these interventions could be attributed to them.

^c^VA builds transmission costs into facility encounter costs.

**Table 4:** Count and prevalence inputs to the model.

Description	Base estimate^a^	Sensitivity estimate^a^
Total SCI patients^b^	20,000	—
New SCI patient^c^	5%	4.5–5.5%
Exiting patient, new PU^d^	25%	22.5–27.5%
Existing patient, flap surgery^c^	1.5%	1.35–1.65%
Existing patient, neither PU nor flap surgery^e^	68.5%	66–71%
Live in catchment area^c^	50%	45–55%
Eligibility for telehealth care		
Lack of functional independence	20%	18–22%
Lack of telephone land line^c^	48%	43–53%
Eligible^e^	32%	39–25%

PU: Pressure ulcer; SCI: spinal cord injury.

^a^All figures are per year.

Sources: ^b^National VA electronic database; ^c^Palo Alto VA electronic database; ^d^Gélis et al. [10]; ^e^authors' calculation.

**Table 5:** Cost estimates by scenario.

	Total cost estimates^a^		
	Low	Medium	High
Scenario 1: Preventive care			
Within SCI homecare catchment area			
Standard care	$19,955	$23,673	$32,933
Telehealth option 1^b^	$18,465	$21,349	$28,669
Outside SCI homecare catchment area			
Standard care	$5,514	$9,242	$24,057
Telehealth option 1^b^	$18,465	$21,349	$28,669
Telehealth option 2^c^	$4,895	$8,066	$14,934

Scenario 2: Existing pressure ulcer			
Within SCI homecare catchment area			
Standard care	$10,100	$11,234	$12,368
(No telehealth in this situation)			
Outside SCI homecare catchment area			
Standard care	$6,795	$8,408	$13,368
Telehealth option 3^d^	$4,901	$6,325	$11,083

Scenario 3: Postsurgical care			
Within SCI homecare catchment area			
Standard care	$4,184	$6,199	$13,769
Telehealth option 1^b^	$7,897	$9,331	$14,093
Telehealth option 3^d^	$2,159	$3,619	$9,515
Outside SCI homecare catchment area			
Standard care	$2,828	$4,719	$12,154
Telehealth option 1^b^	$6,903	$8,161	$12,746
Telehealth option 3^d^	$2,159	$3,619	$9,515

SCI: Spinal cord injury.

^a^Figures are in 2007 dollars.

^b^Telehealth option 1 used an interactive videoconferencing machine that was installed in the patient's home. ^c^Telehealth option 2 used the same machine plus station-to-station (*hub* and *spoke*) teleconferencing. ^d^Telehealth option 3 used low-cost equipment such as digital cameras and e-mail.

**Table 6:** System-level cost implications of standard care versus telehealth.

	Cost estimates^a^		
	Low	Medium	High
Scenario 1: Preventive care, new injuries			
Within SCI homecare catchment area			
Net savings: Usual-telehealth option 1^b^	$214,560 to $262,240	$334,656 to $409,024	$614,016 to $750,464
Outside SCI homecare catchment area			
Net savings: Usual-telehealth option 1^b^	$-1,864,944 to $-2,279,376	$-1,743,408 to $-2,130,832	$-664,128 to $-811,712
Net savings: Usual-telehealth option 2^c^	$89,136 to $108,944	$169,344 to $206,976	$1,313,712 to $1,605,648

Scenario 2: Existing pressure ulcer			
Outside SCI homecare catchment area			
Net savings: Usual-telehealth option 3	$1,550,160 to $1,894,640	$1,704,960 to $2,083,840	$1,870,560 to $2,286,240

Scenario 3: Postsurgical care			
Within SCI homecare catchment area			
Net savings: Standard-telehealth option 1^b^	$-160,402 to $-196,046	$-135,302 to $-165,370	$-13,997 to $-17,107
Net savings: Standard-telehealth option 3^d^	$87,480 to $106,920	$111,456 to $136,224	$183,773 to $224,611
Outside SCI homecare catchment area			
Net savings: Standard-telehealth option 1^b^	$-176,040 to $-215,160	$-148,694 to $-181,738	$-25,574 to $-31,258
Net savings: Standard-telehealth option 3^d^	$28,901 to $35,323	$47,520 to $58,080	$114,005 to $139,339

Total net savings implications based on Telehealth 1 for scenario 1 within SCI CA Telehealth 1 for scenario 1 outside SCI CA Telehealth 3 for scenario 2 outside SCI CA Telehealth 1 for scenario 3 within SCI CA Telehealth 1 for scenario 3 outside SCI CA	$-436,666 to $-533,702	$12,211 to $14,925	$1,780,877 to $2,176,627

Total net savings implications based on Telehealth 1 for scenario 1 within SCI CA Telehealth 2 for scenario 1 outside SCI CA Telehealth 3 for scenario 2 outside SCI CA Telehealth 3 for scenario 3 within SCI CA Telehealth 3 for scenario 3 outside SCI CA	$1,970,237 to $2,408,067	$2,367,936 to $2,894,144	$4,096,066 to $5,006,302

CA: Catchment area; SCI: spinal cord injury; VA: U.S. Department of Veterans Affairs.

^a^Figures are in 2007 dollars.

^b^Telehealth option 1 used an interactive videoconferencing machine that was installed in the patient's home. ^c^Telehealth option 2 used the same machine plus station-to-station (*hub* and *spoke*) teleconferencing. ^d^Telehealth option 3 used low-cost equipment such as digital cameras and e-mail.
